# Trust and Community Treatment Orders

**DOI:** 10.3389/fpsyt.2019.00349

**Published:** 2019-05-20

**Authors:** John McMillan, Sharon Lawn, Toni Delany-Crowe

**Affiliations:** ^1^Bioethics Centre, Dunedin School of Medicine, University of Otago, Dunedin, New Zealand; ^2^Flinders Human Behaviour and Health Research Unit, Flinders University, Adelaide, SA, Australia; ^3^College of Medicine and Public Health, Flinders University, Adelaide, SA, Australia

**Keywords:** community treatment orders, vulnerability, trust, recovery, engagement (involvement)

## Abstract

There are conflicting views about the benefits of community treatment orders (CTOs) for people with mental illness. While there is a significant literature on the coercive nature of CTOs, there is less on the impact that CTOs have upon trust. A recovery-oriented approach requires a trusting therapeutic relationship and the coercion inherent in the CTO process may make it difficult for trust to be built, nurtured, and sustained between workers and patients. Our aim was therefore to examine the role of trust within the CTO experience for mental health workers and patients on CTOs.

**Methods:** We conducted a thematic discourse analysis of 8 in-depth interviews with people who were currently on a CTO and 10 interviews with multi-disciplinary mental health workers in Adelaide, Australia (total N = 18 interviews). The interviews were coded and analyzed with the assistance of a patient representative. The findings reveal the challenges and opportunities for trust within the coercive relationship of a CTO.

**Findings:** We found that patients have diverse experiences of CTOs and that trust or mistrust played an import role in whether or not they found the CTO beneficial.

I can have different kinds of trust: that you will treat me fairly, that you will have my interests at heart, that you will do me no harm. But if I do not trust your word, can I have genuine trust in the first three? If there is no confidence in the truthfulness of others, is there any way to assess their fairness, their intentions to help or to harm? How, then, can they be trusted? *Whatever* matters to human beings, trust is the atmosphere in which it thrives. (1, p. 31)

Bok points out that there are many kinds of trust and that they are fundamental to human relationships and value. As she observes, the principle of veracity, or in other words the obligation to tell the truth and keep your word, is the foundation upon which other forms of trust are built: if you cannot trust someone to tell you the truth and keep to their word, then that undermines the other kinds of trust that you can have in someone. This is a prerequisite for healthy interactions with other human beings in our homes, neighborhoods, and community, and it could be considered a fundamental need—on a par with physical safety.

When we appreciate how central trust is to all human interaction, it raises questions about how and whether trust can thrive within a relationship that was instigated by a Community Treatment Order (CTO). The participants in our study were based in South Australia and reflected upon their experiences of CTOs as they are implemented by that state’s 2009 Mental Health Act (2). Many Commonwealth jurisdictions, such as England and Wales, Canada, New Zealand, and other Australian states, have CTO regimes. While there are differences in the powers that CTOs enable, how they are authorized, and how they fit within other mental health legalization, what they have in common is that they provide a legal authority whereby involuntary mental health treatment can be administered to patients in the community (3). Many jurisdictions have legislation that enables involuntary mental health treatment in the community, although in the United States and some European countries CTOs are called Outpatient Commitment schemes (4, 5).

From a patient perspective, CTOs give mental health workers the power to coerce, and indeed force, them to have treatment that they do not want. This situation creates vulnerability, and while vulnerability may in some contexts provide a foundation for the extension of trust, it can also create power imbalances that make it difficult for trust to thrive. From a mental health worker’s perspective, the fact that a patient is being treated *via* a CTO implies that they cannot be relied upon to comply with proposed treatment, do not understand what their treatment requires, and do not have insight into their illness nor understand why treatment is appropriate. These factors may lead to the conclusion that a patient is “not trustworthy” with respect to treatment or “worthy” of the power to choose freely. While any care plan formulated as part of a CTO should be based around the preferences of the patient, it is an order imposed by the state that provides the power to coerce a patient into receiving treatment in their own home. When we think about how trust forms and functions within our personal relationships, with our friends and family, these seem quite different from how trust functions within a therapeutic relationship that is not chosen.

Rights-based mental health legislation emphasizes the importance of a recovery-oriented focus, meaning that everything done should be done with the end of facilitating the recovery of a patient with a mental illness (6–9). Given that a significant element of recovery from mental illness is restoring the ability to take control of one’s life, there is a tension in being coerced to accept treatment that someone else thinks will aid your recovery. There is, therefore, the paradoxical possibility of restricting a patient’s autonomy *via* a CTO with the therapeutic purpose of nurturing their autonomy. The effectiveness of CTOs in facilitating recovery has also been extensively reported on and some report that there is insufficient evidence for believing they are any better at improving quality of life than voluntary care (10–13).

While “coercion” is an important issue for mental health and was emphasized in the 1960s by antipsychiatrists such as Szasz (14), it is only in more recent years that there has been broader study of its impact and extensive discussion in the literature (15–18). This literature indicates that coercion exists on a spectrum from encouragement and persuasion to threats (17) and that coercion always requires careful and sound justification.

There is also a growing literature on the coercive nature of CTOs (19–22), much of which expresses reservations about whether the coercive nature of CTOs will be helpful to all patients, whether CTOs may, in fact, cause more harm to service and treatment engagement, and whether alternatives to CTOs might be possible and preferable for some patients. However, other qualitative studies have found that the safety net or “safeguarding” elements of CTOs are valuable for some patients (19). Recent qualitative studies from Norway (5, 23) and the UK (24) have confirmed that patients experience CTOs as coercive but found that there is no single patient or health care worker view about CTOs.

One aspect of the CTO process that remains underexplored in the literature is the impact of CTOs on trust between patients and workers. During the coding of 18 qualitative interviews with psychiatrists, community mental health case managers and patients from South Australia, trust emerged as a key theme. This paper explores how discussions of trust were used by the participants to explain their experiences with the CTO process. Based on the research data presented in this paper, we examine how CTOs create particular challenges for the creation and maintenance of trust, which is problematic since trust is an essential component of the empowerment required for a recovery-oriented approach (25).

We found that the different forms of trust that Bok distinguishes featured in the views of some participants: trust in someone’s word, trust that you will be treated fairly, trust that you will do me no harm, and trust that you have my interests at heart. These forms of trust were both present and absent in the interviews, and in the final section of the paper, we explore the implications of these findings by considering theoretical work on the preconditions of trust. We will show how there can be a relationship between vulnerability and the need for trust. At an interpersonal level, trust requires, in addition to veracity, the belief that a person will take into account our interests. We conclude by suggesting that what makes trust possible implies ways forward for those trying to nurture trust within the context of a CTO. Some patients were able to trust partly because they believed that their health care worker was being honest and open with them. Some others enlisted the support of health care workers not involved in the CTO process and that enabled a different perspective upon care. Conversely, some of the patient participants who did not trust their mental health worker thought they were not being spoken to honestly, that their interests were not viewed as that important, and that what they said was not heard or believed.

## Methodology

This paper reports on a subset of findings from a broader qualitative study of patients’ and workers’ experience of CTOs. Qualitative interviews were chosen as the main method of data collection because they allowed deep examination of the understandings of the research participants, and revealed the meanings that participants applied to their experiences of the CTO process. While analyzing the interview data from the broader study, the researchers found that participants frequently referred to trust, a lack of trust, or compromised trust, and the researchers noticed that perceptions of trust appeared to motivate particular types of actions or levels of engagement by patients and workers during the CTO process. This paper explores the dynamics of trust within the qualitative interview data collected from patients and workers.

## Sample and Recruitment

Patient participants were women and men, aged 18 years and over, living in Adelaide, South Australia. All were patients of the State-funded clinical mental health services, currently on a CTO and beyond the first 6 months of the CTO, recruited *via* their mental health community case managers who determined their ability to provide informed consent and not pose any risks during the interview. The researchers provided information about the study in a presentation to the community mental health team who were then asked to identify potential patient participants from their caseloads and provide them with an information sheet and consent form. This method of recruitment may have introduced some bias, with workers perhaps more likely to refer patients who would report positive experiences. However, the data collected from the patients reflected a mix of positive and negative views about a range of topics, as well as a diversity of CTO experiences, so the authors are confident that the findings were not impacted considerably by selection bias. In order to limit the influence of the referring workers, and to facilitate patient autonomy, in most cases, the patient participants contacted the lead researcher independently of the case manager. This also helped to ensure anonymity of their participation. In a small number of cases, the patient participant was happy for the case manager to provide their contact details to the lead researcher. The lead researcher then contacted the patient participant to arrange a time and place to meet to conduct an interview. All patients were advised that they could withdraw at any time during the study and that this would not be divulged to their case managers. The exclusion criteria for the patient interviews were:

- intellectual or cognitive disability that renders the person unable to provide informed consent;
- current suicidality or other risk as determined by the mental health services; and
- case-note alert signifying two-person contact was required.

Overall, nine patients were asked to participate in the research; eight accepted and one declined.

Mental health worker participants were drawn from the professions of psychiatry, nursing, occupational therapy, and social work, that is, they were either community treating doctors or community case managers. The worker participants were all currently employed for 5 years or more. This was important to ensure an established degree of experience and involvement in CTO applications and administration. They were recruited *via* a general email sent to the service’s clinical lead for distribution to staff. The workers all contacted the researcher directly, and information about their participation was not shared with their employer or colleagues. This supported data quality by ensuring that the workers felt able to freely express their views without their employer knowing what views they had expressed. Overall, 10 workers contacted the researcher and all agreed to participate.

Permission for the study was obtained from the clinical and service directors for region’s mental health services. Ethics approval was granted by the SA Heath Human Research Ethics Committee.

## Data Collection

An interview guide informed by a literature review was developed in consultation with a project reference group (see **Box 1**). The lead researcher, who conducted all interviews to ensure consistency, is a mental health patient advocate (someone with direct consumer and family carer lived experience of mental illness) and has over a decade of experience as a mental health worker. A patient with experience of CTOs was a member of the research team from its beginning.

BOX 1 **Interview Guide**

Workers

1. Describe what you think of CTOs for people with mental illness.
  Benefits? Concerns?
2. Describe your own experience of delivering treatment and care to patients on a CTO.
3. What factors do you consider in determining the level of involvement of the person and their decision-making capacity when applying for a CTO and/or providing treatment and care during the time that they are on a CTO?
4. Describe your experience of the Guardianship Board hearing process and of applying for a CTO, or providing input to an application to the Board.
5. What types of support do you provide to patients while they are on a CTO?
6. Are there circumstances that prevent you from providing the support you would like to provide to patients on a CTO? Explain.
7. What do you perceive as the impacts for patients of being on a CTO?
  Benefits? Problems? Impacts for you/the service/others?
8. Are you involved in the development of mental health care plans for patients on a CTO? If so, your experience of these and processes followed? Client copy? How often reviewed? Your perception of what patients think about them?
9. How could MH services improve how they provide support to people on a CTO?
10. Do you have any other comments to make about your experience of providing treatment and care to people on a CTO?

Patients

1. Describe how you came to be on a CTO. How long? Others? Recollections of interactions with mental health staff and Guardianship Board hearing?
2. Describe your experience of receiving contact with MHS since being on a CTO. Case manager? Psychiatrist? Other support people?
3. Describe the level of involvement in making or sharing decisions about your treatment since being on a CTO. Examples? How you felt about this?
4. Describe the level of involvement in making or sharing decision about other parts of your life since being on a CTO. (e.g., Psychosocial support needs). Examples? How you felt about this?
5. What support does the mental health case manager provide to you as part of their contact with you?
6. What do you perceive as the impacts for you of being on a CTO? Benefits? Problems?
7. Do you have a mental health care plan? Your view of it? Have you seen it/got a copy? How often is it reviewed with you? Your involvement in its review?
8. Do you feel that your life has changed since being on a CTO? Why? Why not? If so, what has changed?
9. How could mental health services improve how they provide support to people on a CTO?
10. Do you have any other comments to make about your experience of being on a CTO?

The research was explained, informed consent was confirmed, and a consent form was signed by all patient and worker participants prior to commencement of interviews. For patients, interviews occurred in their home (n = 4), a public location where the patient felt comfortable (n = 2), or the lead researcher’s office (n = 2). All worker interviews (n = 10) occurred in a private office at their service during their usual working hours and at a time convenient for them. All interviews were audio-recorded with consent, and professionally transcribed to ensure accuracy of the data, except for two patients who requested that an audio-recording device not be used. Perhaps coincidentally, these two participants also chose to undertake the interview in a public location away from their home. They were happy for the researcher to collect them from their home and return them there after the interview. Extensive notes were taken during the interviews with these participants. Trust increased during the course of the interviews, and the lead researcher was invited into the patient’s homes following the interviews. Due to the potential to discuss highly sensitive information about their experience of being on a CTO or administering a CTO, participants were offered support to link with existing supports or services (e.g., Case managers for patients or Employee Assistance Program for workers); however, none reported needing this assistance.

Following the interviews, all participants were provided with the opportunity to view and verify the accuracy of the interview transcript/interviewer notes and to further reflect on or edit their comments. Two participants (Jenny and Joan) took the opportunity to review their transcript, and the changes they made were minor, relating mainly to correcting expression and expanding on their comments. One participant, in particular, provided a detailed letter accompanied by extensive artwork to the lead researcher, re-emphasizing the points that they had discussed during their interview. Aspects of the transcripts relating to trust were not edited by any of the participants. The researchers met routinely to discuss the meaning of the data as interviews proceeded. Where possible, these sessions were audio-recorded to capture the dialogue. Reflective notes were made after each interview to capture the context of the interview and to record the interviewers’ observations.

## Data Analysis

Initially, the researchers performed open-coding of four randomly chosen interview transcripts (two patient and two worker transcripts), independently of each other. They then met to discuss and debate their assigned codes to establish an agreed coding framework. At this point, it was determined by all researchers that trust had emerged from the data as a key theme, and trust was included within the coding framework as a focus of the analysis. For the purposes of coding, we viewed trust as “the firm belief in the competence of an entity to act dependably, securely and reliably within a specified context” (26). This concept of trust was used to code all remaining interviews with the assistance of NVIVO 10 software. Following an initial round of open-coding where relevant segments of the patient and worker interviews were grouped under the broad theme of “trust,” selective coding was then applied. This involved close examination of the data to identify key themes in participants’ discussions, to explore how trust or a lack of trust motivated particular actions and thoughts, and to tease apart differences and similarities in how the workers and patients understood trust during their engagement with CTO processes As such, the coding process involved applying a constructionist lens, which helped to understand the meanings applied to trust, and how trust dynamics influenced the interactions between patients and workers (27). Once approximately three quarters of all interviews were coded in this way, the researchers met again to discuss and determine core and sub-themes relevant to the patient and worker interviews. Some of these themes were the same across both sets of interviews, while other themes were relevant only to either the worker or patient interviews, which reflected important differences between the perspectives expressed. The member of the research team with patient experience of CTOs provided critical comments that were incorporated into the coding of the interviews and the interpretation of the data. Once all interviews were coded, the research team met again to finalize the themes. As this was an exploratory study in an area that has not been researched before, we were not aiming for data saturation. Several of the themes that were developed during the analysis have been used as subheadings within the findings section.

## Findings

Trust emerged as a rich and complex theme. It was clear that several patients derived benefits from being on a CTO, and it appears that, for some, such benefits increased their trust in the potential for the CTO, the system, or particular workers to help them. In the following, we summarize the main trust-related ideas that emerged from the coding. Pseudonyms have been used for the patients and workers.

## The Community Treatment Order Being Beneficial and How That Might Increase Trust

A number of patients who found the CTO to be positive commented upon the way in which communication had occurred.

He’s got a way of kind of reflecting things, simple truths back to me; he won’t kind of over-exaggerate a simple truth.

Thomas

This observation resonates with the work of Sissela Bok, who highlighted how feeling that you are being told the truth is the bedrock upon which other forms of trust are built (1). Other patients spoke about how they had confidence that those caring for them would look after their broader needs and also be there if their condition worsened.

C was very good, like, getting my clothes and paying my bills and keeping on top of that.

Joan

You know, knowing that if you do get sick and that you guys can realise hey you’ve gone off your meds and that and you’re getting sick and that, you have that you know hey we can grab you and bring you in and you know get you back and on the right path again.

John

## Forced Consultations: Implications for Trust

Conversely, the CTO process forced some patients to see particular workers even if these workers did not meet the needs of the patients. Mistrust emerged as a key theme during discussions of such forced consultations. Patients reported mistrusting whether the worker they had been forced to see would represent them with honesty and genuine regard for their needs: the inverse of two of Bok’s kinds of trust. An example of such mistrust was expressed during a discussion about communications with the Guardianship Board.

I did not last session but the session before, and I’ve been told I have to see him, and I don’t want to see him. I don’t know why he treats me like that, why he makes false statements about me, and I’d prefer to see Dr Andrews … I’ve written to the Premier again and the Minister of Health and I’ve said, look, Dr Ball is angry at me, he makes vicious cruel statements about me, and nothing’s been done.

Joan

This observation suggests that withholding trust and casting suspicion on the true intent of the worker is one strategy a patient can use to retain some control within an otherwise disempowering situation, where the removal of choice and autonomy may threaten the safety of the patient. The expression of suspicion also appears as a tool that allows the patient to distance herself from the worker and from the version of reality being described by the worker because she considers them potentially unsafe. As such, maintaining distance through withholding trust is about the only strand of control/power left available to the patient.

This highlights the use of trust or mistrust as an expression of control for otherwise disempowered patients within the CTO process and as a tool that patients can use to mediate their interactions with a coercive system. The perception that a worker could not be trusted to tell the truth about the patient was nested within a set of other experiences about the care given while on the CTO.

I don’t understand, I never will. I never will understand why the CTOs, why the false statements, why no counselling, talking to me, why the anger, why the vicious cruel statements - I’ll say that again—but I’ll never understand…

Joan

## Community Treatment Orders and an Environment of Distrust

Some patients experienced what seemed like a near-complete exhaustion of trust in the institution and workers acting under their CTO. They described experiences in which they felt adrift and unable to exert any influence over their care.

It’s a complete breakdown in trust.

I felt exhausted, as if I’d asked for the world.

Didn’t know who to trust. Staff were split over the way I was being treated. Some would support me and others didn’t. They would tell me what they thought about each other and were split opinions over the quality of care I was receiving. One said to me “They treated you like shit”… Re KW—I won’t be in the same room with her on my own. I can’t trust anything she says. She twists my words and actions. I’ll only see her if my friend is also present now.

Jenny

While for some the experience of a CTO was one where they were being coerced into having treatment that they did not want, they viewed communication within that coercive relationship to be important and they would try to initiate it. One patient tried to initiate communication and trust, even if it was just so that the mental health worker understood what the coerced treatment they were offering was like for that patient.

I don’t want her to come, but at the same time I want to be able to be - you know, to be able to tell them how the drug’s affecting me, because they’ve put me on this drug. I think now they have an obligation to listen to me how it’s affecting me since they put it on me and therefore against my will.

Jenny

For some patients, the exhaustion of trust not only was about their relationship with a worker but also was connected with a broader view about the institution that enacts CTOs and the assumptions within it. They viewed the structures behind CTOs as coercive and saw this as a violation of their autonomy over their life and home.

I associate her with, I call it the Mental Sickness Industry. I associate her with that. I associate her with trauma. I had the police come around to my house once and they were hassling me, the Mental - I call it Mental Sickness Industry, that’s what I call it. They were harassing me about taking medication and drugs and stuff because I didn’t get to an appointment because - and basically, she was there and so I associate her with that and I associate her with being violated in my own home. Like, I’m happy to have you in my home because you asked permission, you didn’t force your way in here. You respect my autonomy…

Jenny

There were instances where the coercive nature of CTOs meant that it was hard for patients to trust workers that they otherwise might have trusted. This was because they were perceived to be agents of a coercive authority, and even though they seemed like good people, they could not be trusted.

## Distrust that Workers Know or Understand Them

In addition to the distrust that was instilled by CTOs, some patients did not trust their workers to know them as another human being or understand their experiences.

Because, no Dr Ball—I don’t know why Dr Ball is persevering and determined to put me on a CTO. He doesn’t know me. I haven’t had proper consults with him for a long time, and the consults that I did have with him he says, “I’ll do what I want to do.”

Joan

I just didn’t talk to anyone and the doctors and that they’d have—you know from what I heard, you know they would have a ward round and that and just didn’t know how to get me out of this mute type situation and that’s how I was for a number of years. You know and they tried everything to try and—not because—not scare me or anything, you know they didn’t know how to wake up.

Joan

## Seeking Workers not Involved in the Community Treatment Order

Although CTOs should include a negotiated treatment plan that ensures patients will have access to workers and therapy that will aid their recovery, some patients reported pursuing contact with workers who were outside of the CTO relationship, that is, they were showing a desire and capacity to trust certain individuals and services.

Oh yes, Salvation Army. I went—I go there for support because sometimes I’m short of money, and I know them because I’ve done voluntary work there before, and they had a brochure, ‘Get a referral to a psychologist’, so I took that in and they contacted the psychologists which is near my home, and thank goodness Dr Coombs could see me because I was quite in shock at being locked up, and I would just stay home, I wouldn’t even contact my friends. So they did a referral to my GP and they fitted me in, and then Dr Coombs went on maternity leave so Dr David took over, and every time I go there I talk and talk and talk.

Joan

## No Second Chance at Trust

Trust can be relevant to whether you think that a worker will listen and take seriously what you say. Within the context of a CTO, some patients described how once trust had gone, they would not trust again.

… there wasn’t a second chance at trust because I told you the first time when I was on the medication I didn’t feel that, because I’d heard that Orders could be made and I was threatened with that, but the first person that brought that up is Dr Lee herself in a funny sense, that if I don’t take my medication that an Order can be made to do that, so that builds an instinctive fear so you don’t want to tend to open up to say, ‘It’s not the medication, it’s something to do with my alcohol and nicotine patches,’ and I think maybe in her sense she’s never said to me but she probably doesn’t realise that you can shut somebody from wanting to say more things rather than if you’re threatened with an Order, you know what I mean? And then that’s when you tend to go underground and say, ‘Well I’ll try and do it on my own’ so you have less support because you can’t open up and ask for support.

Vicky

## Worker Perception About who Should be put on a Community Treatment Order

While trust did not feature explicitly in the workers’ experiences of CTOs, they did describe why they thought CTOs were necessary and, in doing so, suggested that there are often problems with the worker–patient relationship because of a lack of insight by the patient. As such, the workers did not trust that patients were fully able to grasp the implications of their illness or what was required to treat their deficits. The implication of this is that some workers seemed oblivious to the impact of the CTO process on the patient’s level of trust in them, and instead interpreted and dismissed patient resistance as an individualized pathology, or personal deficit that could be addressed through expert care.

Complete lack of insight which means that they don’t necessarily see any connection between either their symptoms or the way they’re experiencing things and the need to take treatment and so, in my experience, if there is this complete absence of insight then the use of the CTO does in fact often allow people to almost progress through life without any insight but be reasonably treated and live reasonable lives…

Well if in fact you have a person on your caseload who has a very severe illness, and I will say that in terms of applying for a CTO it is in general only persons who have a very severe illness that I would ever contemplate applying to the Board. For someone who has a mild illness this in general I would not.

*Judith* (Social Worker)

Some mental health workers made observations that suggested that they thought the illnesses being treated *via* CTOs were so serious that they precluded the development of meaningful and trust-based relationships. In effect, the severity of mental illness ruled out “recovery” as an outcome and meant that a smaller step toward mental health was a more realistic goal.

Well, in general, yes, I mean, let’s be real, I mean, why would I wish to go around putting CTOs on people? It’s a huge amount of work, it’s something that you really only do after you have exhausted all possibilities. If during that year people can come to some understanding of their own situation and agree to involve themselves with the medical profession and medication, and also some other recovery sort of type focused actions. I mean, I’ve got one lady on my caseload who I think—this was all before she came onto my caseload—but I think she might have had about five or six CTOs in her time and she was very self-damaging, self-harming, didn’t cope at all but, at one stage she had a series of them and she recovered enough to be able to take control of some of her own medical responsibilities and it worked well.

*Judith* (Social Worker)

While it is clear that CTOs make a patient-centered approach difficult, some mental health workers have found ways to be honest and communicate within a CTO.

Absolutely. Well I mean sometimes you sort of think, say, take my arsonist friend, I didn’t think twice about just saying, ‘Well, this is your opinion, you understand you’ve got an illness but you also state very clearly, and thank you very much for your honesty, but you will not take your medication without it, so we’ll go to the Guardianship Board and we can discuss that there.’

*Judith* (Social Worker)

Despite the honesty expressed by this worker, the power imbalances inherent in the relationship are conveyed through their language. The use of “my arsonist friend” is congenial, but could also be taken to flag the nature of this patient’s problems and to label them as an “arsonist.” Of course, it may be that this patient *is* an arsonist, but this label means that the patient becomes viewed *via* the workers’ interpretation of them. While “friend” in this context is likely to have been used for benevolent reasons, it can also be taken to imply a belittling of the patient.

Views about why some patients are compliant while under a CTO and others not were sometimes justified by features taken to be attributes of the patient and those around them such as friends who may be somehow complicit and untrustworthy too.

CTOs are useful for people who are inherently well socialised and obliging and who are told that they have a legal obligation to participate in treatment. That sub-set of people might be more likely to participate in treatment because of the symbolic value of a CTO but, for people who are determined to evade CTOs and particularly if they have a support network who are able to support them in so doing, CTOs are of limited utility, you’re empowered to inject somebody with medication if they’re in your presence, it doesn’t stop somebody from hiding or running away.

*Tim* (Psychiatrist)

## Engagement Grounded in Trust as an Aim

Some of the mental health workers expressed concerns about the influence that a CTO could have upon their ability to be perceived as trustworthy and authentic, hence to engage effectively with patients. Implicit within such views is the concept of trust: both that the patient will trust what the mental health worker says, their motivation, and their understanding, and that this will result in the worker trusting that the patient will follow through with treatment they have agreed upon. For some mental health workers, the difficulties in establishing a trusting relationship caused by the use of a CTO, and concern about being complicit with the coercive process, were such that they would actively resist their use for some patients.

…but often I’ve disagreed with CTOs because I think a lot of the good work and people wanting to improve their quality of life is down to engagement, and I think if people are given the opportunity to work with somebody they’ve worked with before, then they don’t need a Community Treatment Order, and I think that sounds idealistic in many ways as well…

Absolutely, and it’s really hard and you walk a fine line as well with people on a Community Treatment Order about engagement - really hard. Many a time I’ve refused, out of an engagement perspective, to complete the CTO applications. The doctors have asked me and I’ve said, ‘no, I don’t support the Community Treatment Order and I’m not going to complete the work,’ and the patient wouldn’t even necessarily know that I had actually filled the paperwork in, but I know that I filled the paperwork in and that just seems morally wrong to me when I don’t agree with the Community Treatment Order.

*Laura* (Nurse)

## Discussion

Our participants’ reflections about CTOs illustrated Bok’s point about trust in another’s word as central to all forms of trust. They illustrated the other forms of trust that she mentioned: trust that you will be treated fairly, trust that someone has your interest at heart, and participants also reported on slightly different senses of trust. Being able to trust that a mental health worker will understand or believe what you are saying emerged as an important experience for some patients.

The interviews showed how being required to see a specific mental health worker can result in a loss of patient trust in the worker and the system. While all mental health workers are likely to want their patients to see they are working toward their interests and trust them, as Baier points out, trust does not work by us merely asking or inviting the person to do so.

“Trust me!” is for most of us an invitation which we cannot accept at will—either we do already trust the one who says it, in which case it serves at best as reassurance… (28, p. 244)

Given that workers see themselves as being there to help, it might be thought that patients should and will trust them. However, a trusting relationship needs to be built and sustained, and the first vital step in this process is by being truthful, open, and generous with time in communication. When the therapeutic relationship occurs within a CTO, that creates difficulties for creating and sustaining trust between patient and mental health worker.

It is clear that patients have the capacity to trust and probably would like to be able to trust their mental health worker. But the nature of care within a CTO relationship for many of the patients that we interviewed made this difficult. Some patients sought independently the contact of other workers with whom they developed productive trusting relationships. There is a growing literature on the value of peer specialists (former patients who have experienced recovery) for building empathy and relationships within a mental health team (29). With a good induction to the workplace, peer specialists have been shown to integrate well with the mental health team and improve the experience of patients (30, 31).

Some patients took the coercive nature of the CTO to mean that their worker and the system did not trust them to do what they should be doing. So, from the worker’s perspective, a CTO is a solution to the uncertainty of the future and takes the role that might otherwise be played by trust. Niklaus Lumann observes that there is a conceptual relationship between trust and vulnerability: trust is a way of controlling for the uncertainties that the future holds.

The problem of trust therefore consists in the fact that the future contains far more possibilities than could ever be realized in the present and hence be realized in the past (32).

Intuitively, vulnerability is a crucial concept we associate with any form of treatment for mental illness that occurs within the context of mental health legislations. That is true both in the way that mental health legislation and CTOs can trump the right to refuse medical treatment and in the attempts made by legislators to protect the vulnerability of patients treated under such schemes *via* reviews or appeals. As Lumann observes, vulnerability is important much more generally too in that all of us are vulnerable to the multitude of events that could occur in the future and trust can function as a way of controlling and narrowing down those events. So, as Lumann observes, institutions and generalized systems develop so as to control this vulnerability. Giddens develops similar ideas and shows how trust can bridge the gap between the known and the unknown (33). Within a given societal structure or institution, individuals can initiate the creation of trust, but the rules that govern institutions can also provide a basis for the creation of trust when individuals are unfamiliar with each other.

This analysis raises a question about what happens when a process, such as a CTO, is coercive and counterproductive to the creation of trust. While some processes create trust between unknown actors, is it possible for workers and patient who do not know each other to trust? Because patients have no say in who cares for them *via* the CTO, they may be expected to engage with completely unfamiliar people with whom bonds of trust have been neither formed nor tested. Because of this, patients will base their assumptions about trustworthiness on the processes and system with which they are expected to engage and these assumptions could create additional challenges for creating trust. For this population, there might be added complications and a heightened need to be mindful of these concerns, given that they may be struggling to distinguish reality from delusional thinking when deciding who they can trust.

The CTO process authorizes involuntary treatment, and this means that treatment can be imposed upon patients *via* coercion. While care planning is a requirement for a CTO in most jurisdictions, the rules that govern them are perceived as serving the needs of the system rather than the needs or wants of the patient (34, 35). Therefore, patients may use their experience of this system as the basis for understanding how trustworthy particular individuals are likely to be. The result is that our inherent willingness to trust a mental health worker and service even when they operate in a trustworthy system (governed by standards, ethics, and training) is damaged. CTOs risk creating distrust in a health care system and the actors within this system becoming imbued with the same lack of trustworthiness. So, when trust does grow within the context of a CTO process, it is in spite of this process, as actors have overcome the distrust created by a coercive process.

The way that a CTO controls future possibilities by specifying what will happen if a patient does not comply with the order removes a vulnerability and the need to rely upon trust, risks constructing patients as inherently untrustworthy, and that seems diametrically opposed to a recovery-oriented approach. Nonetheless, that pessimistic conclusion does not take into account that some patients, such as Thomas and John, had more positive experiences of CTOs and believed what their worker said and that they heard him.

If CTOs are to better utilize the ability of patients to trust, then reflection upon the nature of trust in relationships that are not freely chosen seems important. A number of the patients felt distrust because they perceived workers to be too busy to focus upon them and that their worker did not seem to be aware of the significance of having someone in their home and giving them medication that they did not want. Time and open communication that engages and creates space for the patient’s views to be heard seem like ways that this can be improved, even if trust continues to be difficult to establish. It might also be useful to reflect upon what leads us to the judgment that we can trust someone. While trust fulfils a set of particular functions within an institution, its preconditions and function in interpersonal relationships are different.

…trust is not simply a reliance on another to act in a certain way; it involves a belief that for some reason - from self-interest, moral considerations, or affection—we can count on the other to pay attention to us and to our interests. (36, p. 6)

While health care workers treating a patient who is on a CTO have significant power over that patient, and ultimately possess the ability to coerce them into accepting treatment, finding a way to show that they are also motivated to pay attention to the patient and their interests, for reasons other than what is required by the CTO, seems to be of fundamental importance. Perhaps the first step toward interpersonal trust is that which Bok draws our attention to: if we cannot believe what someone says to us, nor that they will do what they say, then it is hard to see how any meaningful interpersonal trust can develop. This process likely works both ways, with workers not believing what the patient on a CTO says, or what they say they will do with regard to “compliance” with treatment. So, a starting point for workers who want to mitigate the challenges created by a CTO is to make sure they take the time to openly, respectfully, and honestly communicate with the patient about their treatment. A mental health care worker who demonstrates their honesty and reliability to a patient might still have to do things that the patient does not want, but at least their reasons will be transparent and they have taken the first and most important step toward creating a trust-based relationship. If that happens, then it might be possible for patients to reciprocate and be frank and open with their health care worker. Under such conditions, trust seems likely to grow and the relationship become more recovery-oriented and more satisfying for the health care worker, as well as better for the patient, as both build hope, through trust, that recovery is possible.

## Conclusions

This paper has illustrated how trust, while complex, can be central to the interactions between mental health workers and patients who are subject to CTOs. While the interviews are qualitatively very rich, because the study involved a small number of participants, it is important to not generalize and make claims about the experiences of all workers and patients involved in CTOs. Nonetheless, we have shown how trust can be difficult to create and sustain within the bounds of a CTO and it would be surprising if others did not have similar experiences. There was a relationship between trust and whether patients found the CTO to be beneficial or harmful. While it will always be challenging to nurture trust within what is a coercive relationship, some of the patients and mental health workers did make this happen and reflecting upon what can help is important. Being sensitive to the power imbalance in such a relationship and what it might mean for interpreting what a patient says and thinks seems key. Mental health care workers will often also need to counteract the experiences that a patient has of the system that they work within and that can create additional interpersonal challenges for the worker.

This study illustrates how mental health workers and patients share a common aim in wishing recovery to be an endpoint of treatment and a CTO. Clearly, some patients were very unhappy with CTOs, but many of their concerns can be framed as the failure of the CTO to be recovery-oriented. For patients who experienced the CTOs as being positive and recovery-oriented, trusting and be trusted by their mental health worker emerged as a key theme. While this is a challenging area for mental health workers, finding ways to build a relationship in which trust can grow makes sense as a way to adopt and recovery-oriented emphasis. However, the basic building blocks on trust can occur within a CTO and focusing upon these might lead to a more recovery-oriented relationship. Creating a humane relationship is fundamental for the skills of health care workers to be effective in helping those mental illnesses work toward recovery. Sissela Bok is correct that trust is the bedrock upon which healthy human relationships are built and that this is as true for CTOs as it is in our other relationships.

## Ethics Statement

This study was carried out in accordance with the recommendations of the “South Australia Health Human Research Ethics Committee” with written informed consent from all subjects. All subjects gave written informed consent in accordance with the Declaration of Helsinki. The protocol was approved by the “South Australia Health Human Research Ethics Committee.”

## Author Contributions

JM, SL, and TD-C all contributed to the writing of this paper. They all saw and approved the paper before it was submitted. SL conducted the fieldwork. SL and TD-C conducted the coding.

## Funding

Flinders University awarded a seeding grant that was used to cover the incidental expenses involved in this research.

## Conflict of Interest Statement

The authors declare that the research was conducted in the absence of any commercial or financial relationships that could be construed as a potential conflict of interest.
